# Cytokines and Alcoholic Liver Disease

**Published:** 1997

**Authors:** Craig J. McClain, Steven Shedlofsky, Shirish Barve, Daniell B. Hill

**Affiliations:** Craig J. McClain, M.D., is chief of the Division of Digestive Diseases and Nutrition at the University of Kentucky Medical Center, Lexington, Kentucky. Steven Shedlofsky, M.D., is chief of the Gastroenterology Division at the Lexington Veterans Affairs Medical Center. Shirish Barve, Ph.D., is assistant professor of medicine and nutrition and Daniell B. Hill, M.D., is assistant professor of medicine at the University of Kentucky Medical Center, Lexington, Kentucky

**Keywords:** cytokines, alcoholic liver disorder, immune response, AODE (alcohol and other drug effects), inflammation, tumor necrosis factor, blood proteins, metabolism, drug theapy, endotoxins, literature review

## Abstract

Chemical messengers called cytokines play an important role during the body’s initial response to infection (i.e., acute inflammation). Cytokines attract and activate components of the immune system, promote blood clotting, and facilitate the release of additional chemical messengers. In addition, cytokines induce the liver to shift its physiological function, emphasizing inflammatory and immune responses at the expense of normal metabolism. Alcohol consumption may cause excessive cytokine production in the liver, leading to inflammatory liver disease. Researchers are seeking ways to moderate the toxic effects of cytokines while sparing their protective functions.

The human body is under constant siege by disease-causing microorganisms. The immune system—a complex network of specialized cells and organs—defends the body against these invaders. Chemical messengers called cytokines play an important role during the body’s initial response to infection (i.e., acute inflammation), when they attract and stimulate immune system cells and orchestrate their interactions. Cytokine levels in the blood normally increase during infection and decrease as infection subsides. Persistently elevated cytokine levels cause symptoms of chronic inflammation, as in alcoholic liver disease (ALD). Researchers are seeking ways to moderate the toxic effects of cytokines while sparing their protective functions. This article explores the relationships among alcohol consumption, cytokine production, and liver disease and discusses implications for the treatment of ALD.

## The Inflammatory Response

The following brief account of inflammatory processes is based largely on the body’s response to infection, which has been extensively studied.^1^ Microorganisms that penetrate the skin or mucous membranes and invade the underlying tissue are confronted by phagocytes, specialized cells that engulf invading microorganisms and cell debris through a process called phagocytosis. The first phagocytes to be activated are macrophages, which reside in tissues and organs throughout the body. Meanwhile, cells damaged by infection release chemicals that trigger additional inflammatory processes. For example, blood-clotting proteins help wall off the injured area, delaying the spread of microorganisms and their toxic products. Local blood vessels become “leaky,” permitting white blood cells to infiltrate the walled-off area. Among the first of these cells to arrive are neutrophils, a type of phagocyte. Monocytes arrive more slowly over days or weeks and develop into macrophages at the site of infection. In addition to performing phagocytosis, these two cell types release substances that attract additional monocytes and neutrophils to the area. Meanwhile, within neutrophils and macrophages, powerful degradative enzymes and toxic oxygen-containing compounds (reactive oxygen species [ROS]) destroy phagocytosed microorganisms.

The increased blood flow, together with the local seepage of blood cells and fluids, contributes to the familiar symptoms of pain, redness, and swelling commonly associated with acute inflammation. Prolonged (i.e., chronic) inflammation is characterized by persistent accumulation of monocytes, accompanied by progressive tissue destruction. Alcohol-induced chronic inflammation of the liver (i.e., alcoholic hepatitis) is characterized by fever, jaundice, and loss of appetite. This condition may progress to alcoholic cirrhosis, which is marked by progressive development of scar tissue that chokes off blood vessels and distorts the normal architecture of the liver (see article by Friedman, pp. 310–316).^2^ Because of the liver’s crucial role in many aspects of bodily function, either condition can be fatal.

Cytokines are the primary chemical messengers involved in both acute and chronic inflammation, attracting and activating phagocytes, promoting the clotting of blood, and facilitating the production of additional chemical messengers, including more cytokines (see table, p. 318 for a list of cytokines discussed in this article). These include four substances known collectively as interleukins (i.e., IL-1, IL-6, IL-8, and IL-10) as well as tumor necrosis factor (TNF) and monocyte chemoattractant protein-1 (MCP-1). A single cytokine may be produced by a variety of cell types in response to different stimuli and may exert multiple effects on different target cells. Conversely, several different cytokines may be able to produce the same effect.

Inflammation is a *nonspecific* immune response, directed against all sources of tissue damage. *Specific* immune responses become dominant when nonspecific defenses are breached. Specific immune responses are typified by the formation of antibodies, proteins designed to recognize and disable specific microorganisms or substances. As the table indicates, cytokines can influence both specific and nonspecific immune responses.

## Alcohol and Cytokine Production

The liver plays a role in the inflammatory response, both as a potential site of chronic inflammatory disease (e.g., ALD) and as a source of phagocytes and cytokines. For example, most of the body’s fixed macrophages reside in the liver, where they are known as Kupffer cells. Kupffer cells assist the liver in screening the blood for infectious or toxic substances picked up from the digestive tract (see sidebar in article by Friedman, p. 311). Alcohol consumption increases the permeability of the intestine, permitting certain toxic bacterial products (i.e., endotoxin) to pass through the intestinal wall into the bloodstream. Upon reaching the liver, endotoxin stimulates the Kupffer cells to produce cytokines, which may contribute to subsequent liver inflammation.

Heavy alcohol consumption activates certain detoxifying enzymes in the liver. The metabolism of alcohol by these enzymes may generate ROS (see article by Fernández-Checa et. al., pp. 321–324), which also can stimulate cytokine production. Excess cytokine levels generated by these two alcohol-induced mechanisms may unleash biochemical processes that further increase intestinal permeability and ROS generation, resulting in escalating tissue injury and inflammation.

## Metabolic Effects of Cytokines

Several organs participate in the process of inflammation. Chief among these is the liver, which undergoes a marked shift in physiologic function, emphasizing inflammatory and immune system function at the expense of normal metabolism. This metabolic shift can be elicited by cytokines. In studies using both human and animal subjects (Hill et al. 1996), injection of cytokines led to an increase in the body’s overall rate of metabolism, accompanied by specific alterations in mineral balance (e.g., decreased zinc levels) and the processing of fats and proteins (see box, p. 319).

**Biological Activity of Cytokines and ALD Complications**

Many experimental effects of cytokine administration correspond to some of the complications of alcoholic liver disease (ALD). For example, collagen (i.e., the protein that forms scar tissue) aids healing, but excess collagen deposition (i.e., fibrosis) is a hallmark of cirrhosis. Increased permeability of the endothelial tissue lining the insides of blood vessels permits immune system cells to seep into injured tissue. However, uncontrolled leakage of fluids into body parts can lead to a swollen abdomen (i.e., ascites) and swollen lower legs (i.e., edema). A breakdown comparison of cytokine effects with ALD complications is as follows:

**Table:** 

Cytokine Effects	Complications of ALD
Fever	Fever
Loss of appetite	Loss of appetite
Neutrophilia	Neutrophilia
Altered protein metabolism	Altered protein metabolism
Decreased glutathione	Decreased glutathione
Increased metabolic rate	Increased metabolic rate
Decreased serum zinc	Decreased serum zinc
Decreased bone density	Decreased bone density
Collagen deposition	Liver fibrosis
Increased endothelial permeability	Ascites/peripheral edema
Brain wave changes	Disordered intellectual functioning

NOTE: For definitions of terms, see glossary, p. 330.

**Table:** Important Cytokines of the Immune System

Cytokine	Principal Functions
*Inflammatory cytokines*	
Interleukin 1 (IL-1)	Produces inflammatory responses; induces fever; and stimulates growth and differentiation of immune system cells
Interleukin 6 (IL-6)	Promotes maturation of cells that secrete antibodies; acts with other cytokines to stimulate other immune system cells; and stimulates production of mediators of inflammatory responses
Tumor necrosis factor alpha (TNF-α)	Promotes inflammatory responses; stimulates neutrophils and macrophages; induces fever; and induces macrophages to produce cytokines
*Immunoregulatory cytokines*	
Interleukin 10 (IL-10)	Inhibits proliferation of certain immune system cells and promotes proliferation of others; reduces production of inflammatory cytokines; and promotes antibody secretion
*Chemokines*	
Interleukin 8 (IL-8)	Attracts neutrophils to the site of an infection
Monocyte chemoattractant	Promotes monocyte infiltration protein-1 (MCP-1)

The changes in protein synthesis during inflammation are dramatic. Cytokines induce the liver to synthesize a broad spectrum of acute-phase proteins, so called because they are detected in the bloodstream during acute inflammation. This effect is accompanied by a marked decrease in the production of albumin, the major protein in the liquid portion of blood. Disordered protein metabolism is also evident in muscle tissue, where protein degradation leads to decreased muscle mass. The cytokines that initiate these changes may be synthesized in the liver, or they may be released at the site of infection, reaching the liver via the bloodstream. Similar changes in protein metabolism have been elicited experimentally by injection of cytokines into human or animal subjects (Hill et al. 1996).

The metabolic effects of cytokine administration also can be elicited by administration of endotoxin. In one study (Gaetke et al. 1997), injection of a low dose of endotoxin in human subjects was followed by a rapid increase in the blood levels of TNF and IL-6, with concurrent development of fever, increased neutrophil levels in the blood (i.e., neutrophilia), and altered protein and mineral metabolism, as described earlier.

These changes reflect a shift of metabolic priorities to meet the needs of defense. For example, fever stimulates the immune system, and neutrophilia ensures a supply of phagocytes to fight infection. Muscle wasting reflects breakdown of proteins into their component amino acids, which travel through the bloodstream to the liver for reassembly into acute-phase proteins (e.g., fibrinogen, which aids in the clotting of blood). Despite the survival value of these effects, however, their similarity to some of the clinical complications of alcoholic hepatitis is noteworthy.

## Cytokines in ALD

Clinical studies support the role of disordered cytokine function in producing the signs and symptoms of ALD. For example, patients with ALD exhibit high levels of interleukins 1, 6, and 8, as well as TNF and MCP-1. Elevated levels of TNF in blood are correlated with poor prognosis in patients with alcoholic hepatitis. Similarly, levels of IL-6 and acute-phase protein were markedly increased during the initial hospitalization of ALD patients, subsiding as liver function improved (Hill et al. 1992). The level of acute-phase protein in these patients was similar to that observed when healthy subjects were injected with a low dose of endotoxin.

IL-8 may promote neutrophilia as well as neutrophil infiltration of liver tissue in ALD (Sheron et al. 1993). Similarly, MCP-1 promotes monocyte infiltration. Conversely, production of IL-10 by monocytes is *decreased* in patients with ALD. This cytokine normally helps curb the inflammatory response. Inadequate levels of IL-10 probably contribute to the uncontrolled production of inflammatory cytokines such as TNF.

Studies using laboratory animals complement observations in human patients. Rats chronically administered alcohol frequently exhibit endotoxin in the blood together with increased production of inflammatory cytokines in the Kupffer cells (Kamimura and Tsukamoto 1995; Nanji et al. 1994*a*). These animals also exhibited increased levels of inflammatory cytokines, such as TNF, in their blood and liver. Furthermore, rats chronically fed alcohol and injected with endotoxin develop much more severe liver injury than do rats exposed to endotoxin alone.

## Prospects for Anticytokine Therapy

Over the past decade, researchers have investigated methods for decreasing cytokine activity. Strategies have included developing medications that block cytokine synthesis or that bind to cytokines and inactivate them after their release. In one study, researchers injected alcohol-fed rats with a chemical selectively toxic to Kupffer cells. This treatment significantly reduced alcohol-induced liver injury by inhibiting cytokine synthesis (Adachi et al. 1994). Another study (Honchel et al. 1992) evaluated the anticytokine properties of a prostaglandinlike substance. Prostaglandins are substances widely distributed in the body, some of which appear to be involved in inflammation. Administration of an experimental chemical with prostaglandinlike properties decreased TNF synthesis and mitigated endotoxin-induced liver injury in rats following long-term exposure to alcohol.

The role of endotoxin in the inflammatory response has suggested several lines of inquiry. For example, Adachi and colleagues (1995) reduced liver damage in alcohol-fed rats by administering antibiotics to suppress endotoxin-producing intestinal bacteria. In a similar study, researchers introduced a specific bacterial strain into the gastrointestinal tract of alcohol-fed rats. This bacterium—a species similar to that used in the production of yogurt—releases a substance that suppresses the growth of endotoxin-producing bacteria (Nanji et al. 1994*b*). Administration of this bacterium alleviated liver damage in alcohol-fed rats.

Finally, considerable progress has been made in developing antibodies that can recognize TNF molecules and disable them. Treatment with anti-TNF antibody markedly decreased liver injury in rats chronically administered alcohol in an experimental model of alcohol-induced liver injury (Iimuro et al. 1997). Although this approach has not been applied to human ALD, research with human subjects has shown promise in other serious inflammatory diseases, such as Crohn’s disease (Dullemen et al. 1995) and rheumatoid arthritis (Elliott et al. 1994).

Implementation of anticytokine therapy requires caution. The acute inflammatory response has evolved over millions of years to enhance survival. For example, adequate levels of TNF and IL-6 are critical for liver regeneration (Hill et al. 1996); to inhibit all TNF or IL-6 activity in a patient with severe liver disease could potentially inhibit recovery. An important goal of cytokine research, therefore, is to develop effective strategies to curb overproduction of cytokines while preserving their beneficial effects.

## Footnotes

1 Inflammation can also occur in response to tissue damage caused by heat, radiation, cancer cells, or toxic chemicals such as alcohol.

2 Alcoholics may develop cirrhosis without manifesting earlier symptoms of hepatitis.
